# A Peptide Derived from Nectin-4 Increases Cisplatin Cytotoxicity in Cell Lines and Cells from Ovarian Cancer Patients’ Ascites

**DOI:** 10.3390/cancers17050901

**Published:** 2025-03-06

**Authors:** Kristin L. M. Boylan, Caitlin Walz, Alexandra M. Schefter, Amy P. N. Skubitz

**Affiliations:** 1 Department of Laboratory Medicine and Pathology, University of Minnesota, Minneapolis, MN 55455, USA; boyla002@umn.edu (K.L.M.B.);; 2 Ovarian Cancer Early Detection Program, University of Minnesota, Minneapolis, MN 55455, USA; 3 Department of Obstetrics, Gynecology, and Women’s Health, University of Minnesota, Minneapolis, MN 55455, USA; schefter@umn.edu; 4 Masonic Cancer Center, University of Minnesota, Minneapolis, MN 55455, USA

**Keywords:** ovarian cancer, spheroids, cell aggregation, Nectin-4, peptides, ascites, cell adhesion, chemotherapy

## Abstract

Background/Objectives: New approaches to the treatment of women with ovarian cancer are desperately needed, since most women develop resistance to chemotherapy and the 5-year survival rate remains low. The hypothesis guiding this study was that the inhibition of cell adhesion could be used as a novel strategy to increase the chemosensitivity of ovarian cancer cells. Methods: The Nectin-4 peptide N4-P10 was used to inhibit the formation of cell–cell aggregates (spheroids) using cell lines and cells isolated from ovarian cancer patients’ ascites. Cell lines were pre-treated with peptide N4-P10 or control scrambled peptides and monitored for spheroid formation with live-cell imaging by digital time-lapse photography. Cells were then tested for the cytotoxicity of the chemotherapeutic agent, cisplatin. Results: Peptide N4-P10 blocked aggregation in cell lines with different levels of Nectin-4 expression and different spheroid morphologies. The cytotoxicity of cisplatin increased in cells pre-treated with peptide N4-P10. Similarly, when single cells were isolated from the ascites of ovarian cancer patients, peptide N4-P10 blocked cell aggregation and increased the cytotoxicity of cisplatin. Conclusions: These results suggest that targeting the cell–cell adhesive property of cancer cells could serve as a new approach to augment the cytotoxic effect of chemotherapy and potentially reduce disease recurrence in ovarian cancer patients.

## Simple Summary

Despite an initial response to chemotherapy, most ovarian cancer patients will relapse within 5 years of diagnosis, and many will become resistant to standard chemotherapy. The aim of this study was to test the inhibition of cell adhesion as a novel strategy to increase the chemosensitivity of ovarian cancer cells. Our previous work on the cell adhesion protein Nectin-4 showed that peptides from the extracellular region of Nectin-4 block the formation of cell–cell aggregates known as spheroids. In this study, we tested the ability of the peptide N4-P10 to block spheroid formation in cell lines and cells isolated from the ascites of ovarian cancer patients using digital time-lapse photography of live cells. Cells were then tested for the cytotoxicity of the chemotherapeutic agent, cisplatin. We found that treatment with peptide N4-P10 blocked aggregation and increased the cytotoxicity of cisplatin in cell lines and patient cells, supporting the efficacy of this approach.

## 1. Introduction

Ovarian cancer is the most lethal gynecological malignancy, resulting in over 14,000 deaths annually in the U.S. [1]. Although most ovarian cancer patients will respond to initial treatment with surgery and treatment with a combination of platinum and taxane, 20–30% of patients will relapse within 6 months [2]. These sobering results highlight the need for strategies to increase the effectiveness of chemotherapy and prevent recurrence.

Ovarian cancer is unique in that the primary route for metastasis is intra-abdominal. Fluid is recruited into the abdominal cavity (ascites fluid) and tumor cells released from the primary tumor exist in a free-floating form as single cells and multicellular aggregates, or spheroids, which subsequently attach and invade the mesothelial cell lining [3]. This adhesive attribute and the unique route for metastasis of ovarian cancer cells lend themselves to the possibility of targeting cell adhesion as a novel therapeutic strategy when used concomitantly with intraperitoneal chemotherapy [4,5].

Cell adhesion is also an important component of spheroid formation [3]. Spheroids are less sensitive to chemotherapeutic agents, in part due to the protection afforded by their multicellular shape, but also due to their slower proliferation rate [3,6]. Spheroid formation is also a characteristic of cancer stem cells, which are chemo-resistant cells that are thought to contribute to tumor recurrence [7,8,9]. These characteristics make spheroids an attractive target for therapy.

The protein Nectin-4 is expressed on the surface of a variety of epithelial cancer cells, including ovarian cancer cells, and plays a role in cell–cell adhesion [10,11]. Interactions between nectins on the surface of neighboring cells are thought to be the first step in the formation of adherens junctions, followed by the recruitment of cadherin [12]. Nectin-4 expression has also been shown to influence cell proliferation and migration, and promote anchorage-independent viability [12,13,14,15,16,17,18,19]. In previous studies, we showed that Nectin-4 was involved in the aggregation of ovarian cancer cells and their subsequent formation into spheroids [13,20]. Furthermore, we showed that synthetic peptides derived from the extracellular domain of Nectin-4 and its binding partner Nectin-1 inhibit cell adhesion and spheroid formation in vitro [13,20]. To determine whether the inhibition of cell adhesion could be used as a novel strategy to increase chemosensitivity in ovarian cancer treatment, we used the Nectin-4 peptide N4-P10 to block spheroid formation in cell lines and cells isolated from ovarian cancer patient ascites and tested them for the cytotoxicity of the platinum chemotherapeutic agent, cisplatin.

## 2. Materials and Methods

### 2.1. Cell Culture

The human cell lines NIH:OVCAR5 (Judah Folkman, Harvard University, Boston, MA, USA) and CAOV3 (Robert C. Bast Jr., MD Anderson Cancer Center, Houston, TX, USA) were grown in complete medium (RPMI 1640 medium containing 10% fetal bovine serum (FBS) for NIH:OVCAR5 cells or DMEM containing 10% FBS for CAOV3) at 37 °C in a humidified incubator with 5% CO_2_, as previously described [20]. Cell lines were authenticated by STR fingerprinting (M.D. Anderson Cell Authentication Core Facility, Houston, TX, USA). The cell line OV2008 was received in 2006 from Barbara Vanderhyden (University of Ottawa, Ottawa, ON, Canada). STR fingerprinting revealed these cells as the cervical cancer cell line ME-180 [21], which is how they are referred to in this manuscript. ME-180 cells were grown in RPMI 1640 medium with 10% FBS.

### 2.2. Patient Sample/Ascites Processing

Samples of ascites fluid from patients diagnosed with stage III or IV high-grade ovarian carcinoma, were obtained through the University of Minnesota Bionet Tissue Procurement Facility with approval of the University of Minnesota Institutional Review Board. Ascites fluid was collected at the time of debulking surgery, prior to chemotherapy. Cells were collected by centrifugation, as previously described [22]. Aliquots of cells were suspended in 90% FBS and 10% dimethyl sulfoxide (DMSO; Sigma-Aldrich, St. Louis, MO, USA), and then stored in liquid nitrogen. The patient sample numbers used in this study correspond to the patient sample numbers assigned in our previous publications [13,22,23].

### 2.3. Peptide Synthesis

Peptide N4-P10 (QARLRLRVLVPPLP) and scrambled control peptides, N4-P10S1 (RPVQLALPRLVRPL) and N4-P10S2 (PRRVLPQLALPLVR), were synthesized by Aapptec (Louisville, KY, USA), as previously described [13]. The two scrambled versions of peptide N4-P10 were designed by randomly reordering the 14 amino acids present in peptide N4-P10 so that all three peptides contained the same 14 amino acids, but in a different order for the “scrambled” peptides. The peptides were synthesized with a free amino terminus and an amide at the carboxy terminus (CONH2) and purified by high-pressure liquid chromatography to >95% purity. Lyophilized peptides were stored at −20 °C and dissolved in DMSO at a concentration of 50 mg/mL immediately before use.

### 2.4. Flow Cytometry

Cells were labeled with monoclonal antibodies (R&D Systems, Minneapolis, MN, USA) against Nectin-4 (MAB2659, clone #337516) and Nectin-1 (MAB2880, clone #610835) or isotype controls (MAB0041, Clone #133303; MAB0031, Clone #133304) in a three-step process, as previously described [13]. Patient samples were passed through a 40 µm cell strainer to remove large aggregates prior to staining. LIVE/DEAD™ Fixable Aqua viability stain (ThermoFisher, Waltham, MA, USA) was used to exclude dead cells from the analysis, according to the manufacturer’s instructions. After labeling, samples were fixed in 1% formaldehyde in PBS. Data were acquired at the University of Minnesota Flow Cytometry Resource with a BD FACSymphony A3 flow cytometer (BD Biosciences, Franklin Lakes, NJ, USA) and analyzed with FlowJo software (Version 10.10.0). Cells were gated with Forward Scatter and Side Scatter, single cells and viability. The Fluorescence Minus One-APC control was used to set the threshold for positivity.

### 2.5. Spheroid Formation Assay

Adherent cell lines were detached using Accutase cell dissociation buffer (BioLegend, San Diego, CA, USA) and plated in complete media at a concentration of 5000 cells/well in 96-well, round-bottomed, ultra-low-attachment plates (Corning, Corning, NY, USA) in the presence of 400 µg/mL peptide N4-P10, or 400 µg/mL of two different sequences of scrambled peptides (N4-P10S1 and N4-P10S2). An equivalent volume of DMSO was used as a control. The final peptide concentration of 400 µg/mL was determined in optimization assays to provide the robust inhibition of spheroid formation for an assay time of up to 72 h (Supplemental Figure S2). The concentration of peptide N4-P10 was kept constant for all assays, in order to quantify the LD_50_ of cisplatin, and facilitate comparisons among cell lines and patients’ ascites samples. Bright field and phase contrast images were collected at 1–2 h intervals using the IncuCyte SX5 instrument (Sartorius, Göttingen, Germany) in the University of Minnesota Imaging Center, using the single spheroid module with a 10X objective (software versions 2021C and 2024A Rev2). Spheroids were allowed to form for 24 to 72 h before the addition of cisplatin (AdipoGen^®^ Life Sciences, San Diego, CA, USA) diluted in PBS. Three independent experiments were run on different days, with 8 technical replicates per treatment in each experiment.

For patient samples, cryopreserved cells isolated from the ascites of patients with ovarian cancer were thawed and passed through a 40 µm cell strainer to remove large aggregates. Single cells were plated in RPMI 1640 containing 10% FBS at a concentration of 5000 cells/well in the same manner as the cell lines. Two or three independent experiments were run on different days, with 8 technical replicates per treatment in each experiment.

### 2.6. Cell Viability

Cell viability was determined using the CellTiter-Glo^®^ 3D reagent (Promega, Madison, WI, USA) according to the manufacturer’s instructions. To reduce interference between wells, cells were plated in black/clear 96-well, round-bottomed, ultra-low-attachment plates (Corning). Luminescence was measured in the University of Minnesota Cytokine Reference Laboratory in a Synergy LX multi-mode plate reader (Bio Tek, Winooski, VT, USA) with default settings. Viability for each peptide treatment was calculated as a percentage of the average luminescence in the wells without cisplatin. Prism [GraphPad; version 10.1.1 (270)] was used to plot the viability versus the concentration of cisplatin and determine LD_50_ using nonlinear regression. Statistical significance was determined using the Compare option in Prism, which uses the extra sum-of-squares F test to compare the LD_50_ values generated by the nonlinear regression models.

## 3. Results

Three cell lines with different spheroid morphologies and different levels of Nectin-1 and Nectin-4 expression were used to assess the ability of the Nectin-4 peptide N4-P10 to inhibit spheroid formation over a period of 24 to 72 h. After the spheroids were fully formed, the spheroids were treated with the chemotherapeutic agent cisplatin for 24 h and then tested for cytotoxicity.

### 3.1. Effect of Nectin-4 Peptide on Cisplatin Cytotoxicity of NIH:OVCAR5 Cell Line

**Figure 1:**
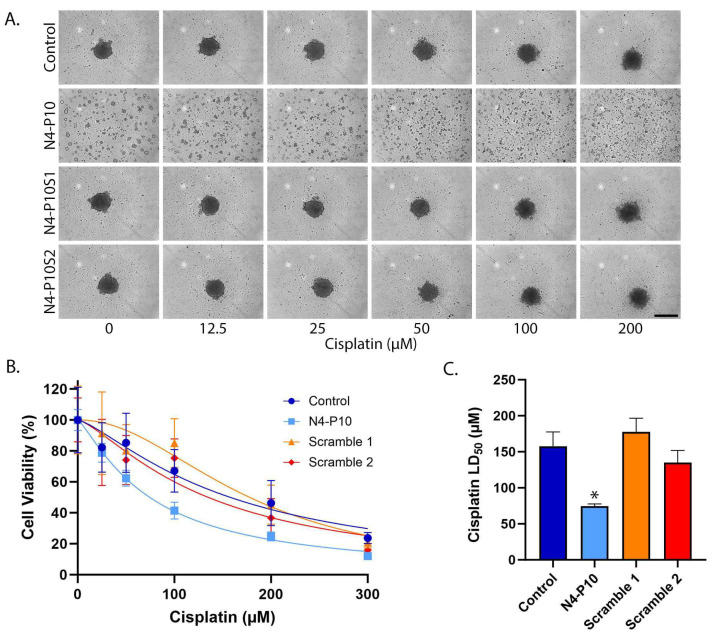
Nectin-4 peptide N4-P10 inhibits spheroid formation and increases cisplatin sensitivity in NIH:OVCAR5 cells. (**A**) Representative images of NIH:OVCAR5 spheroids 24 h after treatment with increasing concentrations of cisplatin. Scale bar = 400 µm. (**B**) Cell viability was plotted against the cisplatin concentration to determine the LD_50_ for cisplatin in the presence of peptide N4-P10, N4-P10 scrambled peptides (N4-P10S1 and N4-P10S2), or DMSO (control). The graph shown is the average of three independent experiments; each experiment had eight replicates per treatment. Luminescence readings were normalized against the cell viability in wells without cisplatin (plotted as 100% viable). The average luminescence of the blank defined 0% viability. Nonlinear regression was used to determine LD_50_ with 95% confidence intervals. Error bars = SD. (**C**) The average LD_50_ for cisplatin for NIH:OVCAR5 cells pre-treated with peptide N4-P10, N4-P10 scrambled peptides (Scramble 1 and Scramble 2), or control (DMSO), calculated from the nonlinear regression analysis shown in B. Error bars = 95% confidence intervals (* *p* < 0.05).

The first cell line tested was the ovarian cancer cell line NIH:OVCAR5. We have previously shown that NIH:OVCAR5 cells express moderate levels of Nectin-4 protein and low-to-moderate levels of Nectin-1 protein on their cell surface [13,24]. When plated under low attachment conditions, NIH:OVCAR5 cells form tightly contracted spheroids within 24 h, in a process that is mediated by Nectin-4 cell adhesion and can be disrupted by treatment with the Nectin-4 peptide N4-P10 [13,20]. To determine whether the inhibition of spheroid formation affects sensitivity to cisplatin, NIH:OVCAR5 cells were plated in the presence of 400 µg/mL peptide N4-P10, two different sequences of scrambled peptides (N4-P10S1 and N4-P10S2), or DMSO (control). Cells were incubated for 24 h to allow cell aggregation and then treated with increasing concentrations of cisplatin (Figure 1). NIH:OVCAR5 cells formed a single, large, tight spheroid in wells treated with either of the two N4-P10 scrambled peptides or just DMSO (control). In contrast, in wells plated with peptide N4-P10, only very small aggregates formed. After 24 h of treatment with increasing concentrations of cisplatin, the tight spheroids that formed in the control and scrambled peptide wells were somewhat larger and looser than the wells without cisplatin treatment, suggesting that cisplatin was killing the outer layer of compacted cells. In contrast, the small aggregates formed in the presence of peptide N4-P10 almost completely disaggregated and appear to be single cells (Figure 1A). Cell viability was measured in each well using the CellTiter-Glo^®^ 3D reagent. Cell viability as a function of the cisplatin concentration was used to calculate the LD_50_ with 95% confidence intervals (CI) for cisplatin treatment under each of the four conditions (Figure 1B,C). The LD_50_ values for DMSO and the scrambled peptides ranged from 135 µM to 178 µM cisplatin (Figure 1B). In contrast, cells treated with peptide N4-P10 exhibited a significantly lower LD_50_ of 75 µM cisplatin. In three replicate experiments, the average LD_50_ cisplatin values for cells pre-treated with peptide N4-P10 decreased by 52.7% compared to cell treated with only DMSO or the scrambled peptides.

### 3.2. Effect of Nectin-4 Peptide on Cisplatin Cytotoxicity of CAOV3 Cell Line

**Figure 2:**
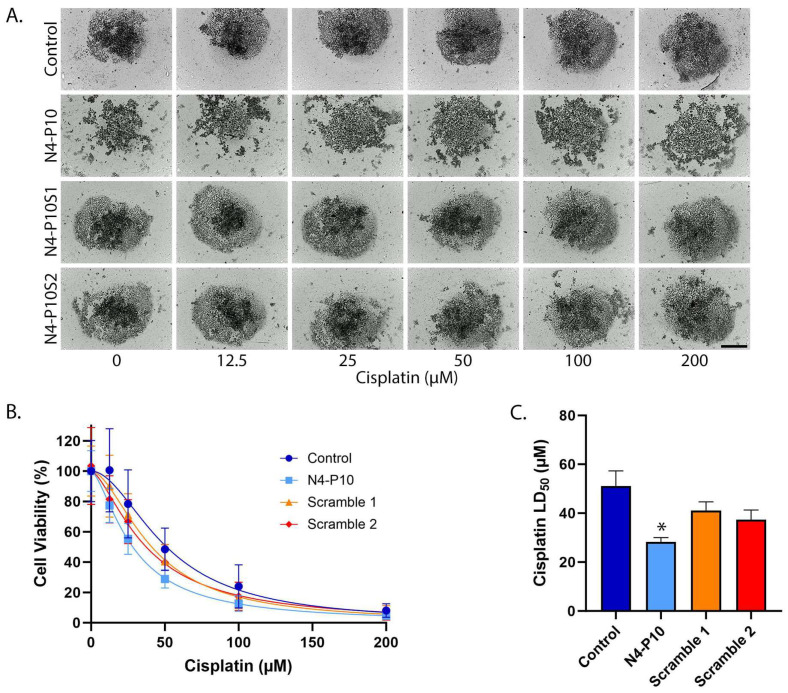
Nectin-4 peptide N4-P10 inhibits spheroid formation and increases cisplatin sensitivity in CAOV3 cells. (**A**) Representative images of CAOV3 spheroids 24 h after treatment with increasing concentrations of cisplatin. Scale bar = 400 µm. (**B**) Cell viability was plotted against the cisplatin concentration to determine the LD_50_ for cisplatin in the presence of peptide N4-P10, N4-P10 scrambled peptides (N4-P10S1 and N4-P10S2), or DMSO (control). The graph shown is the average of three independent experiments; each experiment had eight replicates per treatment. Luminescence readings were normalized against the cell viability in wells without cisplatin (plotted as 100% viable). The average luminescence of the blank defined 0% viability. Nonlinear regression was used to determine LD_50_with 95% confidence intervals. Error bars = SD. (**C**) The average LD_50_for cisplatin for CAOV3 cells pre-treated with peptide N4-P10, N4-P10 scrambled peptides (Scramble 1 and Scramble 2), or control (DMSO), calculated from the nonlinear regression analysis shown in B. Error bars = 95% confidence intervals (* *p* < 0.05).

The second cell line tested was the ovarian cancer cell line, CAOV3, which expresses moderate levels of Nectin-4 protein but very low levels of Nectin-1 protein [13,24]. The majority of CAOV3 cells formed compact, irregularly shaped aggregates (spheroids) within 72 h of plating in round-bottom, ultra-low-attachment plates [20]. Similarly to NIH:OVCAR5 cells, the spheroid formation of CAOV3 cells was blocked by treatment with peptide N4-P10, but not by N4-P10 scrambled peptides [20]. We tested whether blocking spheroid formation with peptide N4-P10 would increase the sensitivity of CAOV3 cells to cisplatin treatment compared to control spheroids or spheroids formed in the presence of scrambled peptides (Figure 2). CAOV3 cells were plated under low attachment conditions in the presence of DMSO (control), peptide N4-P10, and scrambled peptides (N4-P10S1 and N4-P10S2), and spheroids were allowed to form for 72 h. In the presence of scrambled peptides or DMSO (control), tight aggregates formed and were surrounded by a halo of loosely aggregated cells. In contrast, cells treated with peptide N4-P10 did not aggregate or formed only loose aggregates (Figure 2A). A 24 h treatment with increasing concentrations of cisplatin caused the spheroids to fall apart, presumably due to cell death. At the end of the experiment (96 h), cell viability was measured and the LD_50_ with 95% CI for cisplatin was calculated for each condition (Figure 2B,C). The LD_50_ values for DMSO or scrambled peptides ranged from 37 µM to 51 µM cisplatin. In contrast, cells pre-treated with peptide N4-P10 had a significantly lower LD_50_ of 28 µM cisplatin. On average, pre-treatment with peptide N4-P10 decreased the LD_50_ for CAOV3 cells by 44.7% compared to control in three replicate experiments.

### 3.3. Effect of Nectin-4 Peptide on Cisplatin Cytotoxicity of ME-180 Cell Line

**Figure 3:**
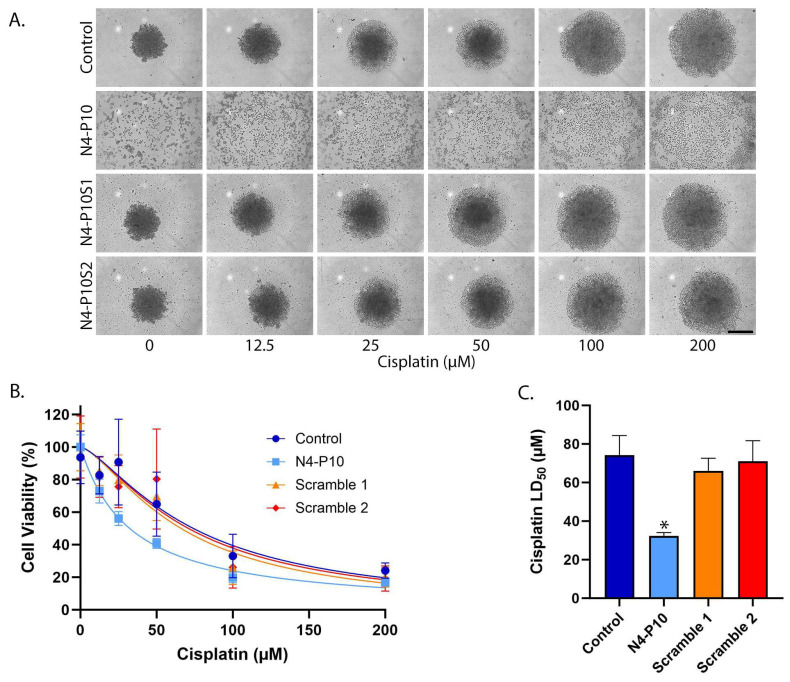
Nectin-4 peptide N4-P10 inhibits spheroid formation and increases cisplatin sensitivity in ME-180 cells. (**A**) Representative images of ME-180 spheroids 24 h after treatment with increasing concentrations of cisplatin. Scale bar = 400 µm. (**B**) Cell viability was plotted against the cisplatin concentration to determine the LD_50_ for cisplatin in the presence of peptide N4-P10, N4-P10 scrambled peptides (N4-P10S1 and N4-P10S2), or DMSO (control). The graph shown is the average of three independent experiments; each experiment had eight replicates per treatment. Luminescence readings were normalized against the cell viability in wells without cisplatin (plotted as 100% viable). The average luminescence of the blank defined 0% viability. Nonlinear regression was used to determine LD_50_ with 95% confidence intervals. Error bars = SD. (**C**) The average LD_50_ for cisplatin for ME-180 cells pre-treated with peptide N4-P10, N4-P10 scrambled peptides (Scramble 1 and Scramble 2), or control (DMSO), calculated from the nonlinear regression analysis shown in B. Error bars = 95% confidence intervals (* *p* < 0.05).

The ME-180 cell line was the third cell line tested for spheroid formation. The ME-180 cell line expresses moderate levels of Nectin-4 and Nectin-1 proteins on the cell surface, as quantified by flow cytometry staining (Supplemental Figure S1A). ME-180 cells were plated with and without 400 µg/mL N4-P10 (Supplemental Figure S1B). Incubation with peptide N4-P10 almost completely blocked the aggregation of the ME-180 cells, even after 48 h (Supplemental Figure S1B). In contrast, ME-180 cells that were plated in the presence of the control (DMSO) or N4-P10 scrambled peptides (N4-P10S1 and N4-P10S2) began to aggregate within 12 h of plating; by 48 h, the aggregates had contracted into large, relatively compact spheroids. The spheroids formed by ME-180 cells were compact, but not as tightly contracted as the spheroids formed by NIH:OVCAR5 cells. We next tested whether peptide N4-P10 would increase cisplatin sensitivity of the ME-180 cells, as it had for the other two cell lines. Cells were incubated for 48 h to allow the formation of compact spheroids, and were then treated with increasing concentrations of cisplatin. After 24 h of cisplatin treatment, the relatively compact spheroids formed in the presence of just DMSO and scrambled peptides were much larger and looser than those spheroids in wells without cisplatin treatment, showing cisplatin’s cytotoxic effect (Figure 3A). Similarly, the small aggregates formed in the presence of peptide N4-P10 almost completely disaggregated and appeared to be single cells after treatment with cisplatin (Figure 3A). The LD_50_ with 95% CI for the cisplatin treatment of ME-180 cells is plotted in Figure 3B,C. The LD_50_ values for DMSO or scrambled peptide ranged from 66 µM to 74 µM cisplatin, whereas the cells pre-treated with peptide N4-P10 had a significantly lower LD_50_ of 32 µM cisplatin. On average, the LD_50_ cisplatin values for cells pre-treated with peptide N4-P10 decreased by 56.3% compared to cells treated with DMSO or the scrambled peptides in three replicate experiments.

### 3.4. Effect of Nectin-4 Peptide on Spheroid Formation of Ovarian Cancer Patients’ Ascites Cells

To ascertain whether treatment with peptide N4-P10 is a feasible strategy for enhancing chemosensitivity in ovarian cancer patients, a series of experiments were performed using cells from the ascites of four patients with stage III high-grade serous ovarian cancer (HGSOC). A summary of clinical characteristics for the patients is shown in Supplemental Table S1. We selected two patients (#2 and #5) with a short overall survival time (19–37 months) and two patients (#3 and #11) with a longer survival time (over 60 months to 109 months). All the patients underwent primary debulking surgery and standard chemotherapy with platinum and taxane. One of the patients (#5) had suboptimal debulking (more than 1 cm residual disease) and very high pre-treatment CA125 levels (2800 units), while the other three patients were optimally debulked and had lower CA125 levels (85–850).

**Figure 4:**
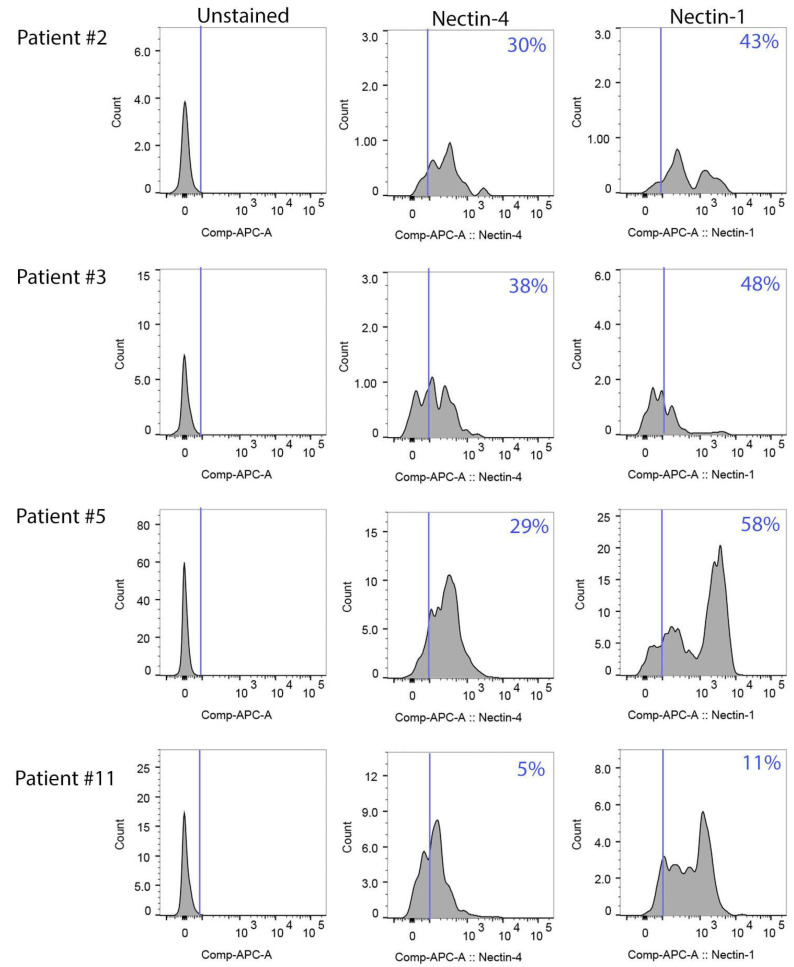
Patient ascites cells express cell surface Nectin-4 and Nectin-1. The cell surface expression of Nectin-4 and Nectin-1 was determined by flow cytometry. The blue line in each histogram marks the threshold for positive staining set by the unstained (Fluorescence Minus One-APC) control. The percentage of viable, single cells that stained positive for Nectin-4 or Nectin-1 is shown in the upper right-hand corner of each graph.

We used flow cytometry to determine the levels of Nectin-4 and Nectin-1 on the surface of the ascites cells. The majority of the single cells (77–90%) were viable as determined by LIVE/DEAD™ Fixable Aqua viability stain. Patient samples expressed varying levels of Nectin-4 and Nectin-1 (Figure 4). Overall, the cell surface expression of Nectin-1 was higher than for Nectin-4; from 11% to 58% of viable cells expressed Nectin-1 compared to 5–38% of cells staining positive for Nectin-4. Patient #5 had the highest percentage of cells that expressed Nectin-1 (58%), and patient #3 had the highest percentage of cells that expressed Nectin-4 (38%). In contrast, patient #11 had the lowest percentage of cells that expressed nectins; only 5% of the cells stained positive for Nectin-4 and only 11% of the cells stained positive for Nectin-1.

**Figure 5:**
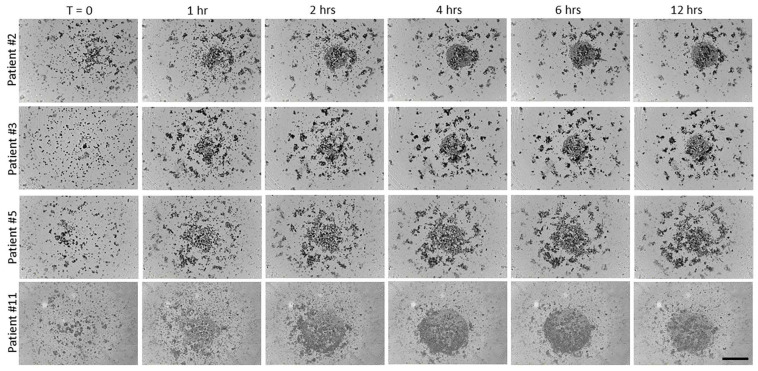
Cells that were isolated from ovarian cancer patient ascites fluid form aggregates in vitro. Single cells isolated from the ascites of four ovarian cancer patients were plated at 5000 cells/well in 96-well round-bottomed ultra-low-attachment plates and monitored in the IncuCyte SX5 imaging system. Representative images are shown at 0, 1, 2, 4, 6, and 12 h after plating. A subset of the cells did not form aggregates, as seen by the circular collection of single cells visible at the bottom of the wells. Scale bar = 400 µm.

**Figure 6:**
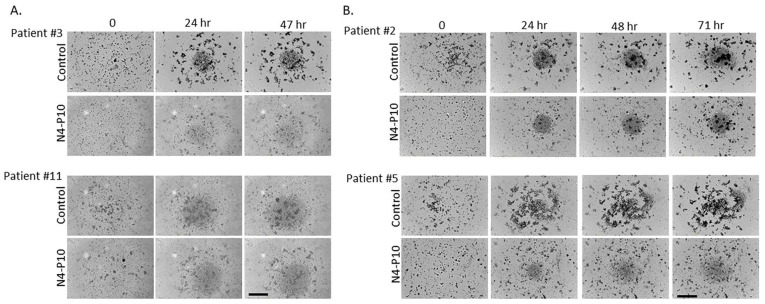
Nectin-4 peptide N4-P10 inhibits the aggregation of ascites cells from ovarian cancer patients. Representative images of the time course of aggregation in ovarian cancer patient ascites cells in the presence of 400 µg/mL peptide N4-P10 or an equivalent volume of DMSO (control). (**A**) Samples from patients #3 and #11 formed tightly aggregated spheroids after 48 h of incubation when pre-treated with DMSO, but not when pre-treated with peptide N4-P10. (**B**) Samples from patients #2 and #5 formed tightly aggregated spheroids after 72 h of incubation when pre-treated with DMSO, but not when pre-treated with peptide N4-P10. A subset of the cells did not form aggregates, as seen by the circular collection of single cells visible at the bottom of the wells. Scale bar = 400 µm.

Cells isolated from the ascites of patients with HGSOC were passed through a 40 µm cell strainer to remove large aggregates. Single cells were then plated into 96-well round-bottomed ultra-low-attachment plates and monitored in the IncuCyte imaging system for several days. Many of the ascites cells began to form small aggregates within 1 h, which increased in size with continued incubation (Figure 5). Small aggregates were present initially (at T = 0) in some samples (e.g., patient #11); these spheroids also increased in size over time. However, a subset of the ascites cells was unable to form aggregates, as seen by the circular collection of single cells visible at the bottom of the wells. With longer incubation times, the cells from patients’ ascites formed multiple, tight aggregates. The ascites cells of two patients (#3 and #11) formed tight aggregates after 48 h in culture (Figure 6A), while the ascites cells from the other two patients (#2 and #5) required 72 h to form tight aggregates (Figure 6B). When cultured in the presence of peptide N4-P10, the size of the aggregates was visibly smaller for all four patients’ cells, demonstrating that cell aggregation was inhibited by peptide N4-P10 in the patients’ samples (Figure 6).

### 3.5. Effect of Nectin-4 Peptide on Cisplatin Cytotoxicity of Ovarian Cancer Patients’ Ascites Cells

**Figure 7:**
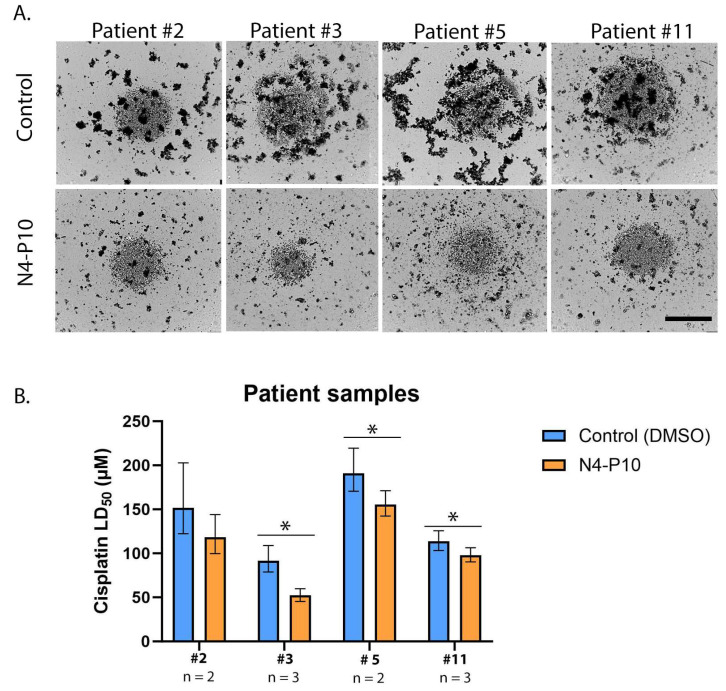
Nectin-4 peptide N4-P10 increases cisplatin sensitivity in ascites cells from ovarian cancer patients. (**A**) Representative images of patient ascites cells cultured with 400 µg/mL peptide N4-P10 or an equivalent volume of DMSO (control). Images were captured after 46 h (patients #3 and #11) or 70 h (patients #2 and #5) of incubation, prior to the addition of increasing concentrations of cisplatin. A subset of the cells did not form aggregates, as seen by the circular collection of single cells visible at the bottom of the wells. Scale bar = 400 µm. (**B**) The average LD_50_ for cisplatin treatment was determined by nonlinear regression for patient samples cultured with 400 µg/mL peptide N4-P10 or an equivalent volume of DMSO (control). The graph is an average of two or three independent experiments; each experiment had eight replicates per treatment. Error bars = 95% confidence intervals (* *p* < 0.05).

Patients’ ascites cells were then tested to determine whether pre-treatment with peptide N4-P10 would increase their sensitivity to cisplatin (Figure 7). Cells were plated at 5000 cells/well in 96-well round-bottomed ultra-low-attachment plates in the presence of 400 µg/mL peptide N4-P10 or an equivalent volume of DMSO (control). The cells were incubated for 48 h (patients #3 and #11) or 72 h (patients #2 and #5) to allow for the formation of compact spheroids (Figure 7A). Increasing concentrations of cisplatin were added and the cells were incubated for 24 h. Cell viability was measured to calculate the LD_50_ with 95% CI for cisplatin for each patients’ cells (Figure 7B). The cisplatin LD_50_ ranged from 92 µM to 191 µM for the patients’ cells treated with DMSO (control) compared to a range of 52 µM to 155 µM cisplatin for patients’ cells treated with peptide N4-P10. Overall, this resulted in an average decrease in cisplatin LD_50_ of 24% in replicate experiments, with a significantly lower LD_50_ (*p* < 0.05) for patients #3, #5, and #11. The LD_50_ for patient #2 also decreased; however, the decrease was not statistically significant (*p* = 0.094).

## 4. Discussion

New approaches to the treatment of women with ovarian cancer are desperately needed, as most women treated with conventional chemotherapy (platinum and taxane drugs) will recur with chemoresistant disease. In this study, we used live-cell imaging to demonstrate that treatment with peptide N4-P10, a 14-amino acid sequence from the extracellular domain of Nectin-4, could inhibit spheroid formation in cell lines as well as cells that were isolated from the ascites of ovarian cancer patients. In addition, cells treated with peptide N4-P10 showed increased cisplatin sensitivity compared to cells treated with a scrambled peptide sequence or DMSO controls, reducing the LD_50_ for cisplatin by more than 50% in cell lines and 25% in ascites cells from ovarian cancer patients.

This study expands upon our previous work in cell lines in two significant ways. First, by increasing the concentration of peptide N4-P10 over what we have previously published [13,20], we found that peptide N4-P10 treatment blocked cell adhesion for at least 72 h in all samples. Secondly, by using cells isolated from ovarian cancer patients’ ascites, we demonstrated that single ascites cells form aggregates ex vivo in a process that appears to be initiated by nectin-mediated cell adhesions.

**Figure 8:**
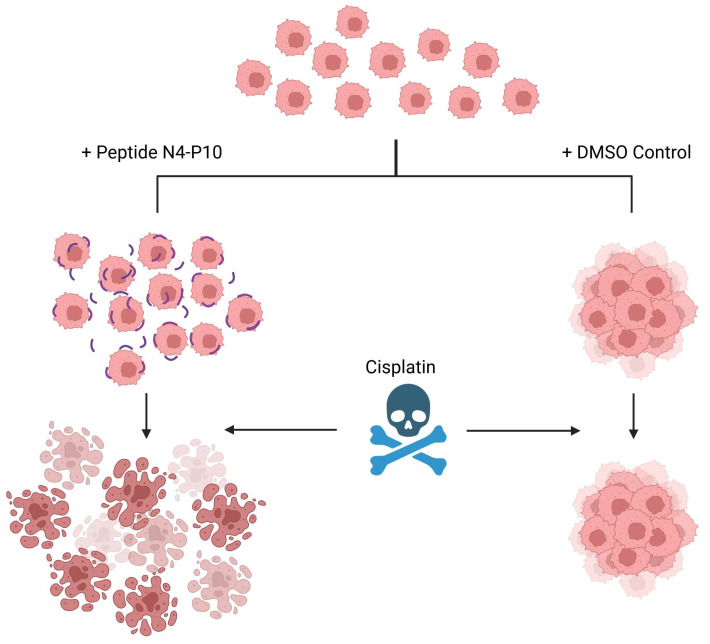
Model for Nectin-4 peptide N4-P10 inhibition of spheroid formation and cisplatin sensitivity in ovarian cancer cells. Single tumor cells shed from the primary tumor into the ascites fluid form aggregates that are resistant to chemotherapy (+DMSO Control). When cells are pre-treated with peptide N4-P10 (purple lines), cell adhesion is inhibited, rendering them more sensitive to cisplatin treatment. Created in BioRender. Boylan, K. (2025) https://biorender.com/q12j146.

We also showed that pre-treatment of cells with peptide N4-P10 resulted in the formation of smaller aggregates and increased the cytotoxicity of cisplatin in cell lines and in cells isolated from the ascites of ovarian cancer patients. In addition, the small spheroids that formed in the presence of peptide N4-P10 were more sensitive to treatment with cisplatin than cells treated with a scrambled peptide sequence or DMSO controls (Figure 8). Similarly to our results, others have shown that treating cells with an antibody to E-cadherin [25] or a laminin-derived peptide [26] in vitro caused the formation of smaller spheroids and increased sensitivity to chemotherapeutics. In contrast, Gunay et al. [27] recently reported that neither spheroid size (120–260 µm) nor morphology (loose aggregates vs. tightly compact spheroids) affected the cytotoxicity of cisplatin in two other ovarian cancer cell lines (OVCAR3 and OVCAR8). Although we did not explicitly test spheroids of different sizes, we found that the very small spheroids formed in the presence of peptide N4-P10 were significantly more sensitive to cisplatin treatment than the large spheroids formed by cells treated with DMSO or scrambled peptides.

When we tested cell lines that expressed different levels of Nectin-4 and Nectin-1 and had different spheroid morphologies, we found that peptide N4-P10 was effective in preventing cell aggregation regardless of the level of nectin expressed, the type of spheroid (loose vs. compact) formed, or the time to spheroid formation. Our previously published studies using ovarian cancer cell lines show that peptide N4-P10 blocks adhesion to the extracellular domain of Nectin-1 in vitro, as do antibodies to Nectin-4 [13]. We have also demonstrated that peptide N4-P10 blocks early steps in spheroid formation [20]. Given that interactions between nectins on the surface of neighboring cells are thought to be the first step in the formation of adherens junctions [11] and that Nectin-4 has been shown to bind to itself and Nectin-1, (but not to other nectins or cell adhesion molecules) [12], it seems likely that peptide N4-P10 functions by blocking these cell–cell attachments during spheroid formation.

The formation of spheroids in the ascites of ovarian cancer patients could occur via the multicellular detachment of cells from the primary tumor, or via the detachment of single cells combined with aggregation [28,29]. Studies using fluorescently labeled cell lines injected into mouse ovaries suggested that the formation of spheroids occurs via the shedding of multicellular clusters, which would be more resistant to anoikis than individual cells [28]. Using live-cell imaging, we observed that individual ascites cells could form aggregates in vitro, suggesting that spheroid formation in patients may occur by the aggregation of single cells in addition to multicellular shedding, though the adhesions formed by Nectin-4 could confer resistance to anoikis by either mechanism. Indeed, Nectin-4 was identified in a screen for genes required for anchorage-independent viability [18], a process that is crucial for the survival of tumor cells in the ascites microenvironment.

One limitation to our study, which will require further investigation, is to understand the exact mechanism by which peptide N4-P10 increased cell sensitivity to cisplatin. While we favor a mechanism where the smaller spheroids resulting from treatment with peptide N4-P10 are more sensitive to chemotherapy because their smaller size allows greater drug penetration, we cannot rule out a signaling mechanism. In breast cancer cells, Pavlova et al. showed that Nectin-4-mediated cell–cell adhesion resulted in the activation of Src family kinase signaling to promote anchorage-independent growth [18]. This suggests that peptide N4-P10 could increase sensitivity to cisplatin by blocking anti-apoptotic signaling pathways. We have previously explored the role of Nectin-4 in various signaling pathways and found that NIH:OVCAR5 cells that express Nectin-4 have elevated levels of phosphorylated ERK1/2, EGFR and beta-catenin compared to NIH:OVCAR5 cells with low levels of Nectin-4 expression [23]. Although the level of phosphorylated Src was not significantly elevated in the Nectin-4-expressing cells, these experiments were conducted in cells grown as a monolayer, not spheroids. The elucidation of the role of peptide N4-P10 in chemosensitivity will require further study.

Ascites fluid contains multiple cell types, including single cells and multicellular spheroids (e.g., cancer cells, mesothelial cells, fibroblasts, and lymphocytes) [30,31]. For our ex vivo assays, the ascites cells isolated from ovarian cancer patients had undergone very minimal manipulation; the cells were filtered to remove large aggregates, but they were not purified or selected for specific cell types. Therefore, the aggregates that formed may have contained multiple cell types and may not have been composed exclusively of cancer cells. In all four patients’ ascites samples, we observed a subset of cells that did not aggregate over time (Figure 5, Figure 6 and Figure 7); the quantity of single cells that collected at the bottom of the wells roughly correlated with the number of cells that did not stain by flow cytometry for either Nectin-4 or Nectin-1. This finding suggests that a subset of the single cells in the ascites samples was most likely not ovarian cancer cells, while those cells that did express the cell adhesion proteins Nectin-4 and Nectin-1 were the cells that contributed to the formation of spheroids.

Our flow cytometry data show that only a subset of the ascites cells expressed Nectin-1 or Nectin-4. Interestingly, the patient whose cells expressed the most Nectin-4 (patient #3; 38% of the cells expressed Nectin-4 on their surface) had the largest decrease in LD_50_ for cisplatin when cells were pre-treated with peptide N4-P10, decreasing by almost 50% compared to the cells pre-treated with DMSO. In contrast, patient #11 had the lowest percentage of cells staining positive for Nectin-4 (5%) and Nectin-1 (11%), and the largest number of non-aggregating cells in our spheroid formation assays (Figure 5). In addition, patient #11 also had the smallest reduction in cisplatin LD_50_ when pre-treated with peptide N4-P10 (13%). Taken together, these results suggest that the efficacy of pre-treatment with peptide N4-P10 may be at least in part dependent on the level of Nectin-4 expression on the ovarian cancer cells.

Other studies have used cells isolated from ascites to test resistance to chemotherapy [32,33]. Chen et al. [32] made patient-derived “organoids” from small (38 to 100 micron) multicellular spheroids isolated from patient ascites cultured in basement membrane extract for 3–4 days prior to drug treatment. When treated with carboplatin, the IC_50_ values for the organoid cultures ranged from 18 to 99 µM, slightly lower than what we found for cisplatin in our ex vivo aggregates. Velletri et al. [33] cultured a single cell per well in ultra-low-attachment plates and watched for the development of spheroids for a period of 8 to 12 days, which they then propagated in spheroid culture. When they tested the cultured spheroids with carboplatin (up to 75 µM), they found differing levels of sensitivity in different cultures derived from the same patient, likely reflecting the heterogeneity of the patient tumor cells, as the cultures were monoclonal. The use of ascites fluid in culture may have stimulated cell division under low attachment conditions [33]. Although we incubated patient cells for only 2–3 days, it is possible that the spheroids that were formed in our experiments were due (at least in part) to the proliferation rather than the aggregation of single cells. We used fetal bovine serum (FBS) in our media, not ascites fluid (which, as Velletri et al. concluded, stimulates proliferation) [33]. Our previous work showed that peptide N4-P10 blocked adhesion to the recombinant Nectin-1 extracellular domain, which supports a mechanism of cell adhesion vs. proliferation [13]. Others have shown that treatment with a recombinant Nectin-3 extracellular domain can block the aggregation of Nectin-1- and Nectin-3-expressing cells [34], providing further evidence that the formation of spheroids from single ascites cells is due to aggregation rather than proliferation. However, we and others have shown that Nectin-4 also plays a role in proliferation [13,14,15,16,17,18,19], so that possibility cannot be ruled out.

Although we examined only four cases of ovarian cancer, it is interesting to note that the two patients with the longest survival time (patients #3 and #11) had ascites cells that were the most sensitive to cisplatin (i.e., had the lowest LD_50_ values). In contrast, the two patients with the shortest overall survival (patients #2 and #5) had the highest LD_50_ values for cisplatin. Interestingly, cisplatin sensitivity for all the patients increased with pre-treatment with peptide N4-P10. As a corollary, in previous work from our laboratory, we found that the ascites spheroid component of patient #5 exhibited the most invasive characteristics of the eight samples tested [22], indicating the aggressive nature of both ascites components (single cells and spheroids). Others have shown that compact spheroids are more invasive than loose spheroids [35,36]. Perhaps, in addition to increasing chemosensitivity, peptide N4-P10 could inhibit metastasis by preventing the formation of compact spheroids, as well as blocking cell adhesion to mesothelial cells that line the abdominal cavity; however, this is purely speculative.

Nectin-4 overexpression has been reported in numerous types of cancer (reviewed in ref. [37]) including breast, bladder, esophageal, lung, gastric, pancreatic, and prostate [38], in addition to ovarian cancer [24]; high Nectin-4 expression in some tumors was associated with disease progression or poor prognosis [15,17,24,39,40,41,42]. Subsequently, Nectin-4 has been developed as a target for the treatment of solid tumors with the antibody drug conjugate (ADC) enfortumab vedotin [43], which was approved by the FDA for the treatment of urothelial cancer [44,45,46,47,48]. It remains of interest in other cancers that overexpress Nectin-4, including ovarian cancer and triple-negative breast cancer [49]. Other ADCs targeting Nectin-4 are also in development [49,50], as are bicycle (bicyclic peptide) toxin conjugates that target Nectin-4 [51]. However, rather than limiting the cell surface protein Nectin-4 to solely a “traditional” drug target, our data suggest the novel strategy of using a function-blocking Nectin-4 peptide to improve the efficacy of standard front-line chemotherapeutic drugs by inhibiting cell adhesion. This approach is supported by experiments in a breast cancer xenograft model showing that intratumoral injections of an antibody to Nectin-4 can stall tumor growth in vivo by disrupting cell–cell contacts [18]. Furthermore, the identification of Nectin-4 as a central gene involved in peritoneal metastasis in high-grade serous ovarian cancer [52] suggests that peptide N4-P10 may be able to reduce metastatic spread by blocking tumor cell adhesion to the mesothelial cells that line the abdominal cavity.

Intraperitoneal therapy for the treatment of ovarian cancer has been used for more than 30 years [4] as a logical means to kill the ovarian cancer cells locally where they reside, versus intravenous chemotherapeutic treatments that are administered systemically (reviewed in [4,5,53]). Drug delivery systems that are injectable or implantable such as hydrogels, films, nanoparticles, and microdevices can be used to deliver cytotoxic agents within the device and offer the advantage of sustained drug release for extended periods of time [5]. Implantable drug delivery systems that use anti-neoplastic agents, hyperthermia, immunotherapy, photodynamic therapy, and gene therapy have been reported [4,5]. The novelty of using a peptide, such as peptide N4-P10, that blocks cell–cell aggregation and thereby increases the cytotoxicity of cisplatin, has yet to be translated into the clinical setting to improve the chemotherapeutic response for ovarian cancer patients.

## 5. Conclusions

Targeting the cell–cell adhesive property of cancer cells has the potential to serve as a new approach to augment the cytotoxic effect of traditional chemotherapeutic drugs. This is particularly relevant for ovarian cancer, given its intraperitoneal route of metastasis and the use of IP chemotherapy. Our study showed that the treatment of ovarian cancer cells with the Nectin-4 peptide N4-P10 inhibited cell aggregation and increased the cytotoxicity of cisplatin, and has the potential for development into a novel treatment that will increase the effectiveness of IP chemotherapy and prevent tumor recurrence in ovarian cancer patients.

## 6. Patents

Patent 10,907,212, entitled “Inhibitors of cell adhesion” (issued by the United States Patent and Trademark Office on 2 February 2021), includes the Nectin-4 peptide N4-P10 used in this study.

## Data Availability

The original contributions presented in the study are included in the article/Supplementary Materials, and further inquiries can be directed to the corresponding author/s.

## Supplementary Materials

The following supporting information can be downloaded at: https://www.mdpi.com/article/10.3390/cancers17050901/s1, Figure S1: ME-180 cells express Nectin-4 and Nectin-1 and form spheroids that are inhibited by pre-treatment with peptide N4-P10; Figure S2: Increasing concentrations of peptide N4-P10 inhibits spheroid formation for up to 72 h; Table S1: Patient characteristics.

## Abbreviations

The following abbreviations are used in this manuscript:

N4-P10	Nectin-4 peptide 10
N4-P10S1	Nectin-4 peptide 10 scramble 1
N4-P10S2	Nectin-4 peptide 10 scramble 2
IP	Intraperitoneal
FBS	Fetal bovine serum
DMSO	Dimethyl sulfoxide
PBS	Phosphate-buffered saline
LD_50_	Lethal dose 50
CI	Confidence interval
µm	Micron
µM	Micromolar
SD	Standard deviation
CA125	Cancer antigen 125
HGSOC	High-grade serous ovarian cancer
