# Supplementary material for: A Peptide Derived from Nectin-4 Increases Cisplatin Cytotoxicity in Cell Lines and Cells from Ovarian Cancer Patients’ Ascites
Source: Cancers (Basel). 2025 Mar 6;17(5):901. doi: 10.3390/cancers17050901 (PMC11899234; doi:10.3390/cancers17050901)
Supplement: Supplementary file 1 [file cancers-17-00901-s001.zip › cancers-3479897-supplementary.pdf]

Figure S1

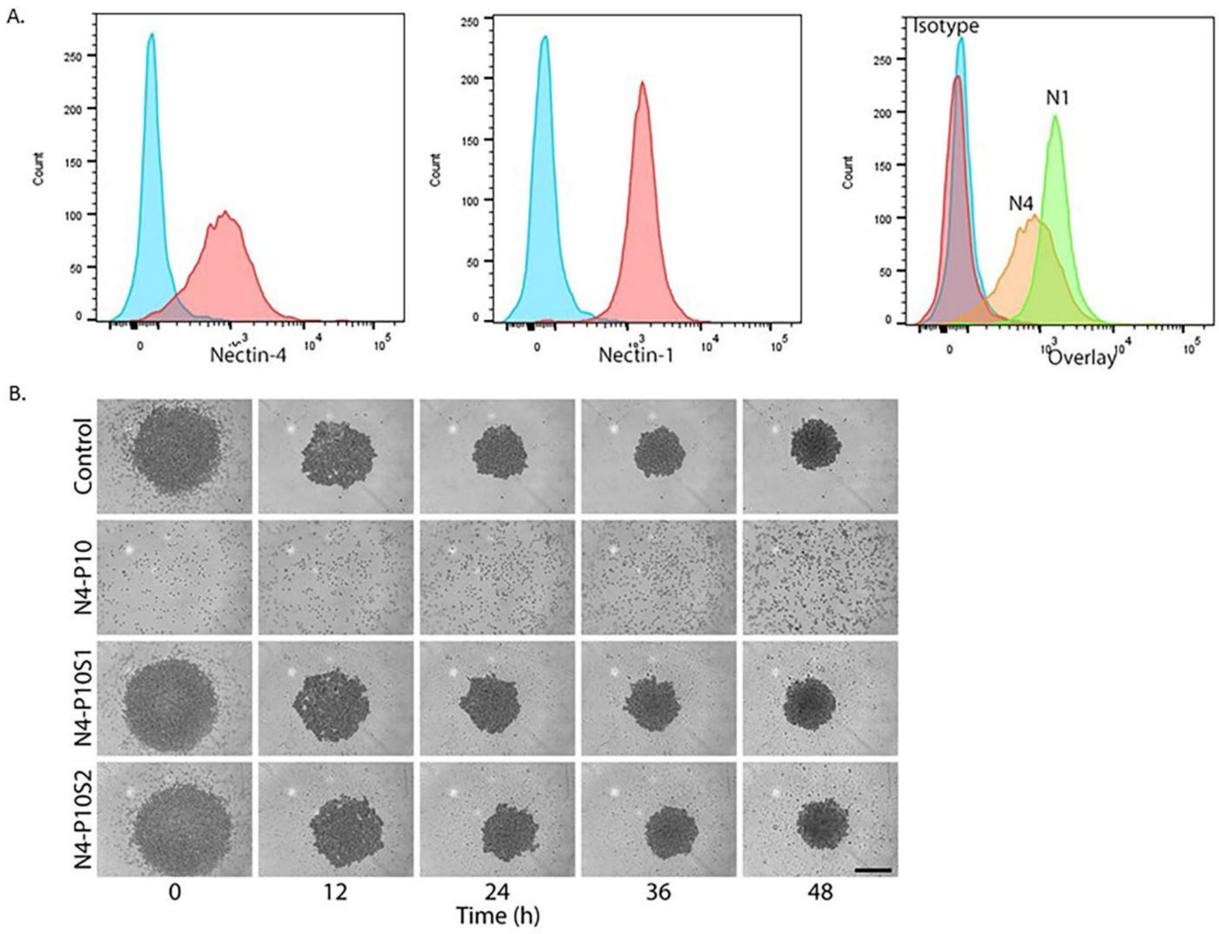

Figure S1. ME-180 cells express Nectin-4 and Nectin-1 and form spheroids that are inhibited by pre-treatment with peptide N4-P10. A) ME-180 cells were analyzed by flow cytometry for the cell surface expression of Nectin-4 and Nectin-1. Shown are histograms for isotype controls (blue) and Nectin-4 (pink, left) and Nectin-1 (pink, center). The panel on the right shows the overlay of Nectin-1 (green) and Nectin-4 (orange) expression. Isotype controls are shown in blue and pink, respectively.

B) Representative images of the 48 hour time course of spheroid formation for ME-180 cells in the presence of 400 µg/ml peptide N4-P10, 400 µg/ml of two different sequences of scrambled peptides (N4-P10S1, N4-P10S2) or an equivalent volume of DMSO (control). Scale bar = 400 µm.

Figure S2

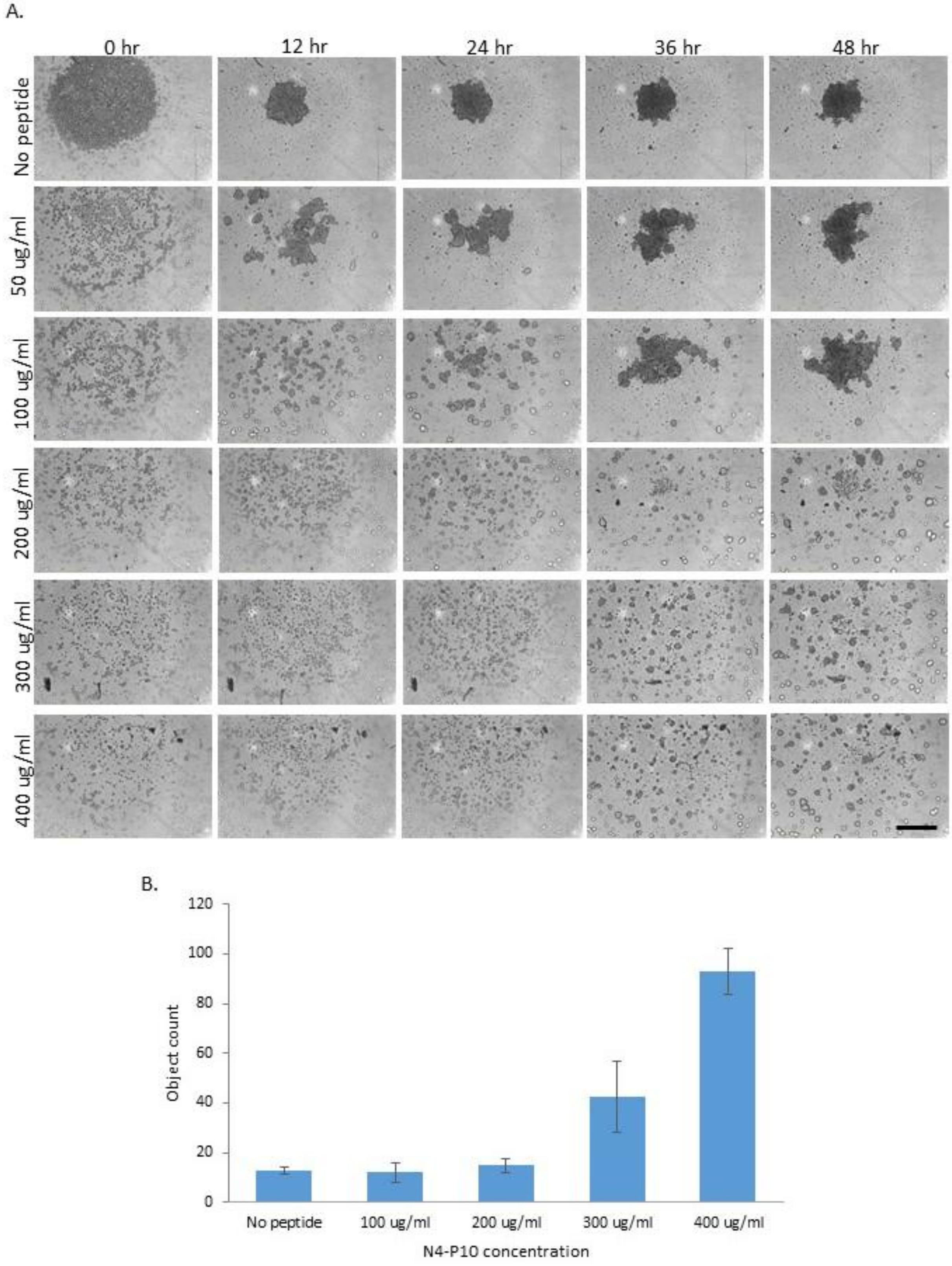

Figure S2. Increasing concentrations of peptide N4-P10 inhibits spheroid formation for up to 72 hours.
A) Representative images of the 48 hour time course of spheroid formation for NIH:OVCAR5 cells in the presence of increasing concentrations of peptide N4-P10. Scale bar = 400 µm. B) In a similar experiment, NIH:OVCAR5 cells were plated with increasing concentrations of peptide N4-P10 and spheroid formation was monitored in the IncuCyte SX5 live cell imaging system for 72 hours. The graph shows the number of objects per image at 72 hours. Error bars = SEM.

Table S1. Patient characteristics

Patient #	Age (years)	Diagnosis	Stage	Grade	CA125	Surgery to Recurrence (months)	Overall Survival (months)	Treatment	Debulking	Ref
**2**	58	High grade serous ovarian carcinoma	IIIC	3	860	22	39	6 cycles Carbo/Taxol	optimal	[22]
**3**	45	High grade serous ovarian carcinoma	IIIC	2	85	36	Alive with disease (>60 months)	6 cycles Carbo/Taxol	optimal	[22]
**5**	70	High grade serous ovarian carcinoma	IIIC	3	2800	Progressive Disease	17	Failed several chemos, Taxol, Carbo, Gemzar	suboptimal	[22]
**11**	64	High grade serous ovarian carcinoma	IIIB	3	544	72	109	6 cycles Carbo/Taxol	optimal	[13, 23]
